# Frontal cortical neuroinflammation and circulating biomarkers following cardiopulmonary bypass in sheep

**DOI:** 10.1186/s40635-026-00986-2

**Published:** 2026-10-10

**Authors:** Taku Furukawa, Alemayehu H. Jufar, Anton L. Trask-Marino, Clive N. May, Roger G. Evans, Andrew D. Cochrane, Bruno Marino, Peter R. McCall, Sally G. Hood, Jaishankar Raman, Connie Pei Chen Ow, Lars I. P. Snel, Scott Ayton, Ashenafi Betrie, Linda Cornthwaite, Munenori Kusunoki, Angela Connelly Huf, Dawoud AlMekhled, Emily Hadzajlic, Adam Southon, Rinaldo Bellomo, Lachlan F. Miles, Lindsea Booth, Yugeesh R. Lankadeva

**Affiliations:** 1 Florey Institute of Neuroscience and Mental Health, University of Melbourne, Parkville, Australia; 2 Florey Department of Neuroscience and Mental Health, University of Melbourne, Parkville, Australia; 3 Department of Critical Care, Melbourne Medical School, University of Melbourne, Parkville, Australia; 4 Cardiovascular Disease Program, Biomedicine Discovery Institute and Department of Physiology, Monash University, Clayton, Australia; 5 Cellsaving and Perfusion Resources, Melbourne, Australia; 6 Department of Anaesthesia, Austin Health, Heidelberg, Australia; 7 Faculty of Medicine, Dentistry and Health Sciences, University of Melbourne, Parkville, Australia; 8 Department of Anesthesiology, Amsterdam University Medical Center, Amsterdam, the Netherlands; 9 Department of Endocrinology, Amsterdam University Medical Center, Amsterdam, the Netherlands; 10 Amsterdam Cardiovascular Sciences Research Institute, Amsterdam, the Netherlands; 11 Department of Anatomy and Physiology, University of Melbourne, Parkville, Australia; 12 Department of Intensive Care, Austin Health, Heidelberg, Australia; 13 Australian and New Zealand Intensive Care Research Centre, Monash University, Clayton, Australia; 14 Department of Intensive Care, Royal Melbourne Hospital, Parkville, Australia; 15 Translational Cardiovascular and Renal Research Group, Stroke and Critical Care Research, The Florey Institute of Neuroscience and Mental Health, 30 Royal Parade, Parkville, VIC 3052 Australia

## Abstract

**Background:**

Cerebral hypoxia and neuroinflammation have been implicated in neurocognitive disorders following cardiac surgery, but their postoperative trajectory and relationship with circulating biomarkers are poorly defined. We investigated cerebral tissue oxygenation, neuroinflammation, and plasma biomarkers of neurodegeneration over 4 weeks after cardiopulmonary bypass (CPB) in sheep.

**Methods:**

Sixteen adult Merino ewes underwent CPB with a 2-h aortic cross-clamp and recovered for either 48 h (*n* = 8) or 4 weeks (*n* = 8). Cerebral tissue oxygenation was monitored. Neuroinflammation was quantified by morphometric analyses of microglia and astrocytes in the frontal cortex in both CPB groups and a naïve control group (*n* = 8). Plasma neurofilament light chain (NfL) and glial fibrillary acidic protein (GFAP) were measured serially.

**Results:**

Compared with pre-CPB baseline, cerebral tissue oxygenation did not change significantly in the 48-h or 4-week period. In the 48-h post-CPB group, microglial soma sphericity was reduced (0.79 ± 0.04 vs. 0.83 ± 0.02, *P* = 0.018) compared with naïve controls, indicating microglial activation. Astrocytic GFAP-positive area was also elevated (17 ± 2% vs. 10 ± 2%, *P* = 0.026). In the 4-week post-CPB group, these measures did not differ significantly from naïve controls. Plasma NfL rose from 138 [96–185] pg/mL at baseline to 198 [149–265] pg/mL at 48 h (*P* = 0.0007), while GFAP peaked at the end of CPB (14.4 [4.7–32.4] vs. 3.0 [1.3–4.5] pg/mL, *P* < 0.0001) and normalised by 48 h.

**Conclusions:**

CPB induced neuroinflammation characterised by frontal cortical microglial activation and astrogliosis, accompanied by temporal elevations in plasma GFAP and NfL. These findings identify microglial activation as a response to CPB in the setting of preserved tissue oxygenation and provide preliminary evidence of an association between perioperative neuroinflammatory changes and circulating biomarkers GFAP and NfL, supporting further investigation.

**Supplementary Information:**

The online version contains supplementary material available at https://doi.org/10.1186/s40635-026-00986-2.

## Introduction

Perioperative neurocognitive disorders are common after cardiac surgery requiring cardiopulmonary bypass (CPB), with delirium occurring in up to 55% of patients [1–3]. These complications are not only associated with short-term morbidity and mortality, but with increased long-term mortality, a higher risk of subsequent dementia, and substantial health care costs [2–6]. The mechanisms underlying these complications are not completely understood, preventing effective targeted therapies from being developed.

Cerebral tissue hypoxia and neuroinflammation are proposed to contribute to cardiac surgery-associated neurocognitive disorders. Clinical studies using near-infrared spectroscopy (NIRS), an indirect measurement of cerebral oxygenation, have demonstrated an association between intraoperative cerebral desaturation and worse neurological outcomes [7–9], while preclinical studies using direct measurements have found cerebral tissue oxygenation is maintained intraoperatively [10–12]. However, cerebral tissue oxygenation in the postoperative period has not been investigated. There is also emerging evidence that neuroinflammation, characterised by activation of microglia [11, 13], may also contribute to CPB-induced neurocognitive disorders [14, 15]. We have previously shown that the frontal cortex is particularly susceptible to CPB-associated neuroinflammation compared with parietal, temporal and occipital cortical regions [13]. Given the established role of the frontal cortex in cognition and executive function, disruption of this region may contribute to development of postoperative delirium. However, whether microglia remain activated in the recovery period following CPB remains unclear. Additionally, while astrocytes also play a critical role in the central response to inflammation and repair [16], the changes in astrocytes in the recovery period following CPB have not been investigated.

Furthermore, blood biomarkers such as neurofilament light chain (NfL – a marker of axonal injury) and glial fibrillary acidic protein (GFAP – a marker of astrocyte injury) are used increasingly as surrogate biomarkers of brain injury and neurodegeneration [17–20]. Perioperative increases in plasma NfL [18–21] and GFAP [22–24] have been reported after cardiac surgery. However, the temporal kinetics of plasma NfL and GFAP have not been systematically investigated before, during and after CPB, and their relationship to histopathological frontal cortical neuroinflammation remains poorly defined.

To address these knowledge gaps, we developed a largemammalian model of CPB with a 4-week recovery period [25]. Using this model, we investigated frontal cortical tissue oxygenation, neuroinflammation (with microglial activation as the primary outcome at 48 h post-CPB), and plasma biomarkers of neurodegeneration, NfL and GFAP, before and during recovery from CPB. We hypothesised that, despite the absence of overt cerebral tissue hypoxia, CPB induces sustained neuroinflammation in the postoperative period, and that such response is accompanied by elevations in blood-based biomarkers of brain injury and neurodegeneration.

## Materials and methods

All procedures were conducted following approval by the Animal Ethics Committee of the Florey Institute of Neuroscience and Mental Health (approval numbers 18-119-FINMH and 22-063-FINMH) and conformed with the Animal Research: Reporting of In Vivo Experiments (ARRIVE 2.0) guidelines [26]. 

A total of 24 healthy adult Merino ewes (1.5–2.0 years of age) were included in this study. Sixteen animals (mean ± SD, body weight: 38.3 ± 3.1 kg) underwent a standardised CPB protocol described below and were assigned to two experimental groups: eight animals were euthanised 48 h after CPB (CPB-48 h group) and eight were euthanised four weeks after CPB (CPB-4w group). The remaining eight healthy animals served as a naïve control group to make immunohistochemical comparisons.

### Surgical preparation

Sheep allocated to the CPB-48 h and CPB-4w groups underwent two aseptic surgical procedures: construction of bilateral carotid arterial loops and implantation of custom-made combination fibre-optic probes (Oxford Optronix, Abingdon, United Kingdom) into the frontal cortex [10]. Animals were allowed at least 5 days to recover following the second procedure. (See Supplementary Material for details.)

### Cardiopulmonary bypass

**Fig. 1:**
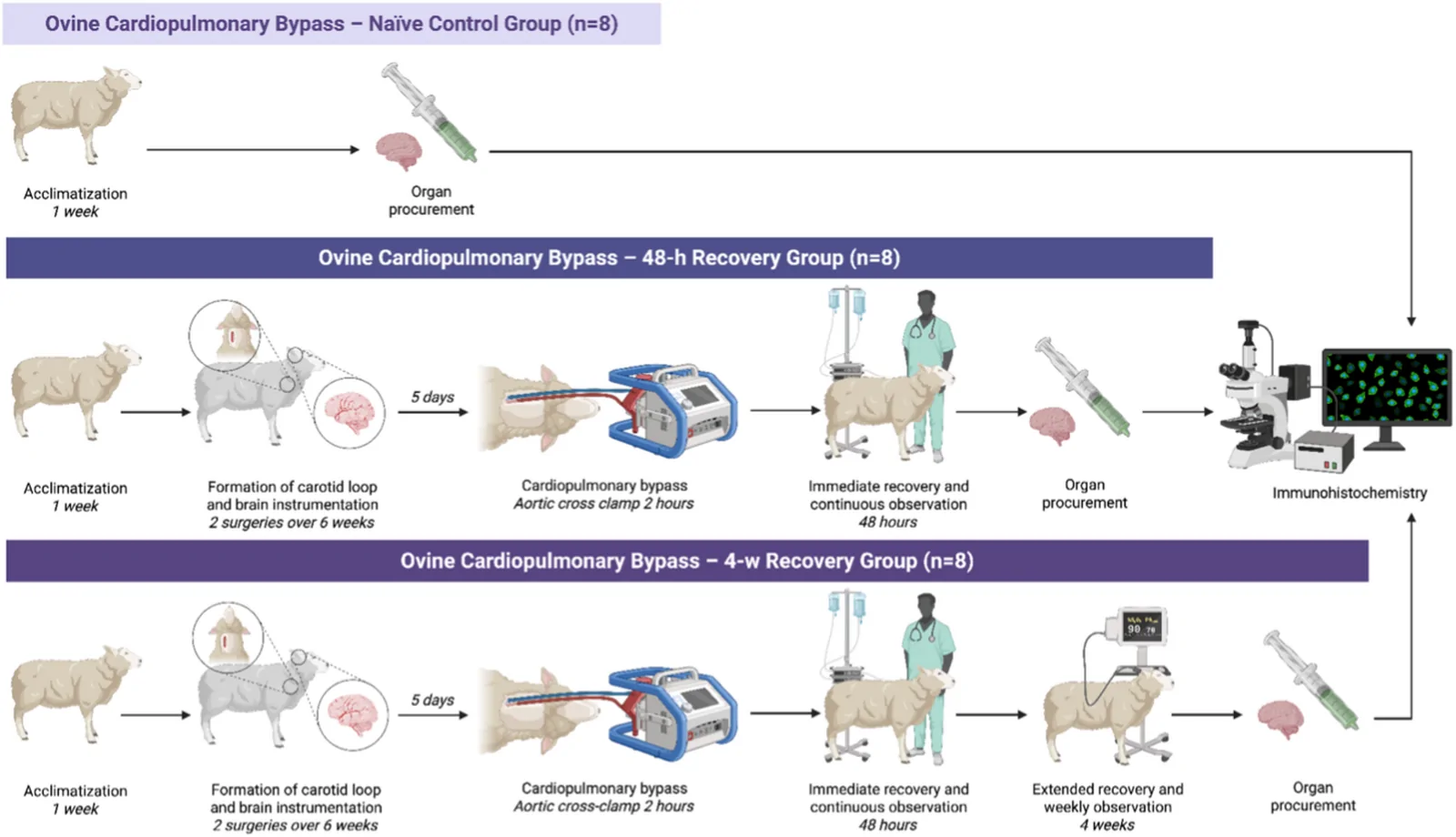
Schematic description of study design

The experimental timeline for CPB and post-CPB monitoring is illustrated in Fig. 1. A detailed description of the CPB procedure is provided in the Supplementary Material. In brief, following a 30-min baseline recording period in the non-anaesthetised state, sheep were transferred to the operating theatre for the CPB procedure. General anaesthesia was induced with intravenous propofol (4 mg/kg, Fresofol, Fresenius Kabi Australia, Australia) and fentanyl (5 µg/kg, Fentanyl GH, Panpharma, Germany) and maintained with a combination of propofol (4 mg/kg/h), fentanyl (5 µg/kg/h) and inhaled sevoflurane (4–5%, Piramal Sevoflurane, Piramal Critical Care, USA). Compound sodium lactate (2 mL/kg/h; Baxter Health Care, Australia) was administered intravenously as maintenance fluid. A right thoracotomy was performed, and a transit-time flow probe (20 mm; Transonic Systems, USA) was placed around the pulmonary artery to measure cardiac output. CPB was then established using previously described methods [11, 25]. We targeted a non-pulsatile flow of 2.4 L/min/m^2^ (body surface area calculated according to Bennett [27]), a mean arterial pressure (MAP) of 65–75 mmHg and a decrease in core body temperature of 3 °C, consistent with current best practice recommendations [28]. Intravenous boluses or a continuous infusion of metaraminol (Montrose Life Sciences, NSW, Australia) were used as required to maintain target MAP. If MAP could not be maintained ≥ 70 mmHg with vasopressors, or if mixed venous SO_2_ was < 70%, pump flow was increased by 10%.

An aortic cross-clamp was applied for 2 h with antegrade blood cardioplegia (4:1; Baxter Healthcare) for myocardial protection. During the final 15 min of the cross-clamp period, animals were rewarmed to 39 °C (basal body temperature for sheep). The aortic cross-clamp was removed, and animals were weaned off CPB. Protamine (3 mg/kg, Fisons Protamine Sulfate, Sanofi, Australia) was administered to neutralise residual heparin. Residual blood from the CPB circuit was infused to optimise volume status. After decannulation and chest closure, anaesthesia was discontinued, animals were extubated when clinically appropriate and then returned to individual metabolic cages for postoperative observation.

### Post-CPB period

After being returned to their cages, the animals were monitored continuously, with all variables recorded for 48 h and averaged hourly. Intravenous infusion of compound sodium lactate was infused at 2–4 mL/kg/h and tapered over 24 h, with 200–250 mL boluses given as needed based on haemodynamic status. Norepinephrine (Medsurge Healthcare, Australia) was infused as needed to maintain MAP > 65 mmHg. Supplemental oxygen was delivered via a nasal cannula at 4.0 L/min, and weaned over 4 h, guided by arterial PO_2_. Postoperative analgesia was provided in accordance with institutional animal ethics approval and standard veterinary care protocols. This included intramuscular flunixin meglumine (50 mg) administered once daily for two days following preparatory surgical procedures and for three days following CPB. In addition, animals received a continuous intravenous infusion of fentanyl (0.5 µg/kg/h) after CPB, which was gradually tapered over 48 h.

Once conscious, sheep had unrestricted access to food and water. The CPB-48 h group was euthanised at 48 h post-CPB. For the CPB-4w group, monitoring was continued for 6 h every week from 1 to 4 weeks post-CPB, with blood samples collected at the end of each monitoring session. Animals in this group were euthanised at the end of the 4-week period. Additional details are provided in Supplementary Material.

### Experimental measurements

Variables, including arterial pressure, cardiac output, cerebral tissue oxygen tension (tPO_2_), perfusion and core temperature, were recorded digitally on a computer using a CED micro 1401 interface and Spike 2 software (Cambridge Electronic Design, Cambridge, United Kingdom) [29]. Blood samples for oximetry, biochemistry (ABLsystem 625; Radiometer Medical, Denmark), plasma cytokine, and brain injury biomarker analyses were collected at baseline, the end of CPB, and at 12, 24, and 48 h postoperatively. In the CPB-4w group, additional samples were collected weekly from 1 to 4 weeks postoperatively as described below. Additional details are provided in Supplementary Material.

### Immunohistochemistry

At the conclusion of the experiment, the animals were euthanised with an intravenous injection of sodium pentobarbital (20 mg/kg; Virbac, Australia). The brain was immediately collected for immunohistochemical assessment as described previously [11]. Briefly, the brain was perfused via the carotid arteries with 1 L of cold saline, followed by 1 L 4% paraformaldehyde in phosphate-buffered saline (PBS). The tissue was then placed in paraformaldehyde for 24-h at 4 °C for further fixation, followed by dehydration in 20% sucrose. Once dehydrated, the brains were cut into 5 mm coronal slices and frozen on powdered dry ice and stored at − 80 °C. Coronal section (40 μm thick) of the frontal cortex were prepared using a cryostat. We selected this region following our previous findings that the frontal cortex is the most susceptible area of the cortex to post-CPB neuroinflammation [13], and its established role in cognition and executive functions, which, when disrupted, likely underpins delirium. Sections were incubated for 48 h with guinea pig anti-ionised calcium-binding adaptor molecule 1 (Iba1; Synaptic Systems GmbH, Germany; 1:1000) and anti-rabbit GFAP (Agilent Dako, United States; 1:2000) to label microglia and astrocytes, respectively. Sections were then incubated for 1 h with Donkey anti-Guinea pig AlexaFluor488 and Donkey anti-rabbit AlexaFluor594 secondary antibodies (Jackson Immuno, 1:500), mounted on slides using gelatin and then cover-slipped with mounting medium containing 4′,6-diamidino-2-phenylindole (DAPI; Sigma-Aldrich, St. Louis, USA). Images were acquired using the Nikon-Crest Spinning Disk Confocal microscope, at 40× magnification (objective Plan-Apochromat, 40×/1.25, silicone), with the system-recommended 0.35 μm step size for a z-stack size of 20.1 μm. Microglial morphology was analysed in Imaris v10.2 (Oxford Instruments, Abingdon, UK) [30]. While resting microglia have a smaller cell body and longer branched processes, activated microglia have larger cell bodies with shorter retracted processes [13, 31]. Astrocytes were analysed using ImageJ (NIH, Bethesda, USA). The percentage area and fluorescence intensity of GFAP was determined using maximum projected images.

### Plasma biomarkers

Plasma concentrations of interleukin-6 (IL-6; pro-inflammatory) and IL-10 (anti-inflammatory) were measured at baseline, at the end of aortic cross-clamp, and at 24 and 48 h post-CPB in both the CPB-48 h and CPB-4w groups using enzyme-linked immunosorbent assay (ELISA; IL-6: DIY1900B Kingfisher Biotech, USA; IL-10: SHFI00009, Assay Genie, Ireland) [11, 13]. In the CPB-4w group, additional measurements were taken at 1, 2, 3, and 4 weeks post-CPB. Plasma levels of NFL and GFAP were quantified using single molecule array (SIMOA) [32, 33] with the Neurology 2-Plex B Advantage Plus Assay Kit (Quanterix, Billerica, USA) on a Quanterix HD-X automated immunoassay analyzer at baseline, the conclusion of aortic cross-clamp, 24 h and 48 h post-CPB in both groups. An additional measurement was performed at 4 weeks post-CPB in the CPB-4w group to assess whether elevations persisted long-term and to coincide with the timing of histological assessment. Additional measurements between 1 and 4 weeks were not performed because the primary focus of the study was the early postoperative period, when neurological complications most commonly occur.

### Statistical analysis

The primary outcome was microglial activation at 48 h post-CPB, assessed by morphometric analysis of microglia in the frontal cortex. Based on our previous study [13], we calculated that a sample size of eight animals per group would provide a 90% power at an α level of 0.05 to detect differences in microglial branch number. To assess longer-term effects, an additional eight animals were allocated to the CPB-4w group. However, due to incomplete tissue preservation during post-mortem processing, only five brains from this group were available for immunohistochemical analyses. Key secondary outcomes included cerebral tPO_2_, astrocytic reactivity assessed by GFAP-stained area, and plasma levels of NfL and GFAP. Histological analyses were performed according to the predefined experimental groups. For physiological and blood biomarker analyses, data from the CPB-48 h and CPB-4w groups were combined up to 48 h after CPB because both cohorts underwent identical procedures and assessments during this period.

All statistical analyses were performed using GraphPad Prism for Windows, version 10 (GraphPad Software, USA). Data are presented as mean ± SD or median [interquartile range, IQR], depending on distribution assessed using the Shapiro–Wilk test. For paired comparisons, either paired t-tests or the Wilcoxon matched-pairs signed-rank test was used. For comparisons involving three or more time points, either mixed-effects modelling with a Greenhouse–Geisser correction or the Friedman test was applied for the main effect of time, as appropriate. If a significant time effect was observed (*P* ≤ 0.05), multiple comparisons versus pre-CPB baseline were conducted using Dunnett’s test or Dunn’s test. For immunohistochemical analyses, a one-way ANOVA was applied, followed by Dunnett’s multiple comparisons between each group. Two-tailed P-values ≤ 0.05 were considered statistically significant.

## Results

### Immunohistochemistry

**Fig. 2:**
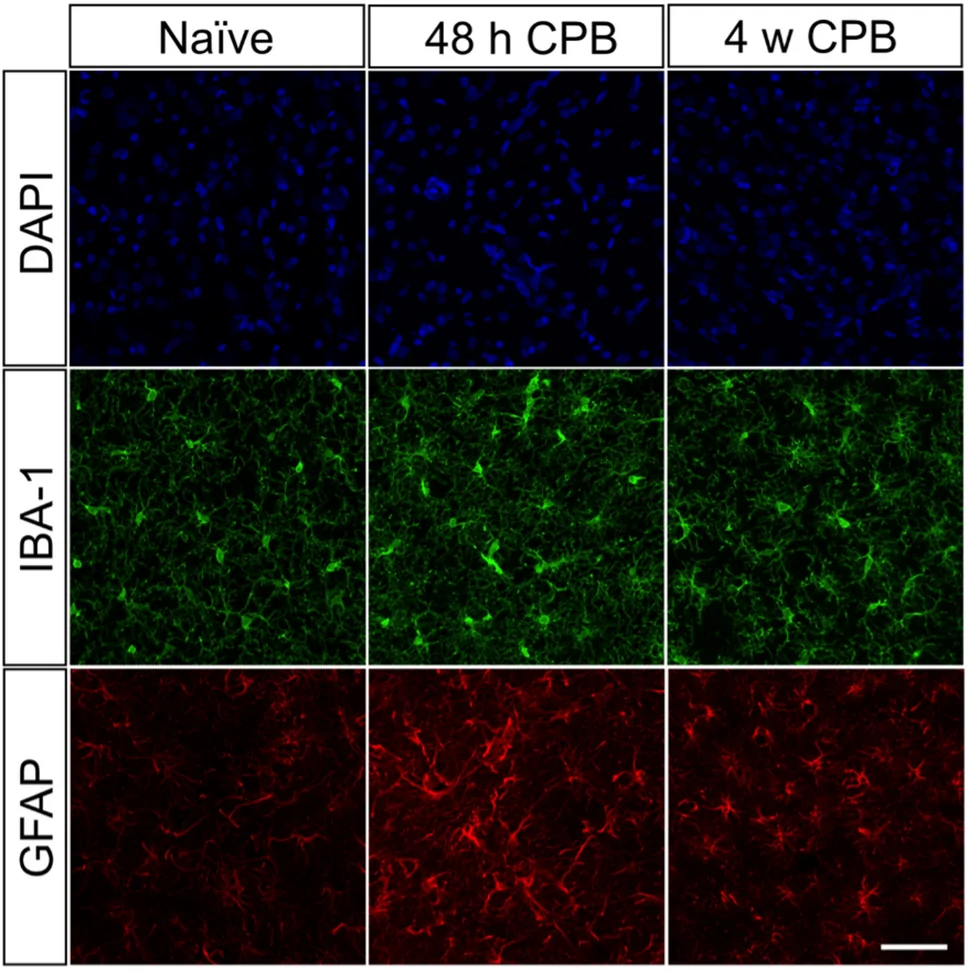
Microglia and astrocytes after cardiopulmonary bypass (CPB). Representative images from the frontal cortex of naïve sheep, sheep 48 h after CPB (48 h CPB) and sheep 4 weeks after CPB (4 w CPB) showing nuclei (4’,6-diamidino-2-phenylindole (DAPI); blue), microglia (ionised calcium-binding adaptor molecule-1 (IBA-1); green) and astrocytes (glial fibrillary acidic protein (GFAP); red). Scale bar represents 50 μm

**Fig. 3:**
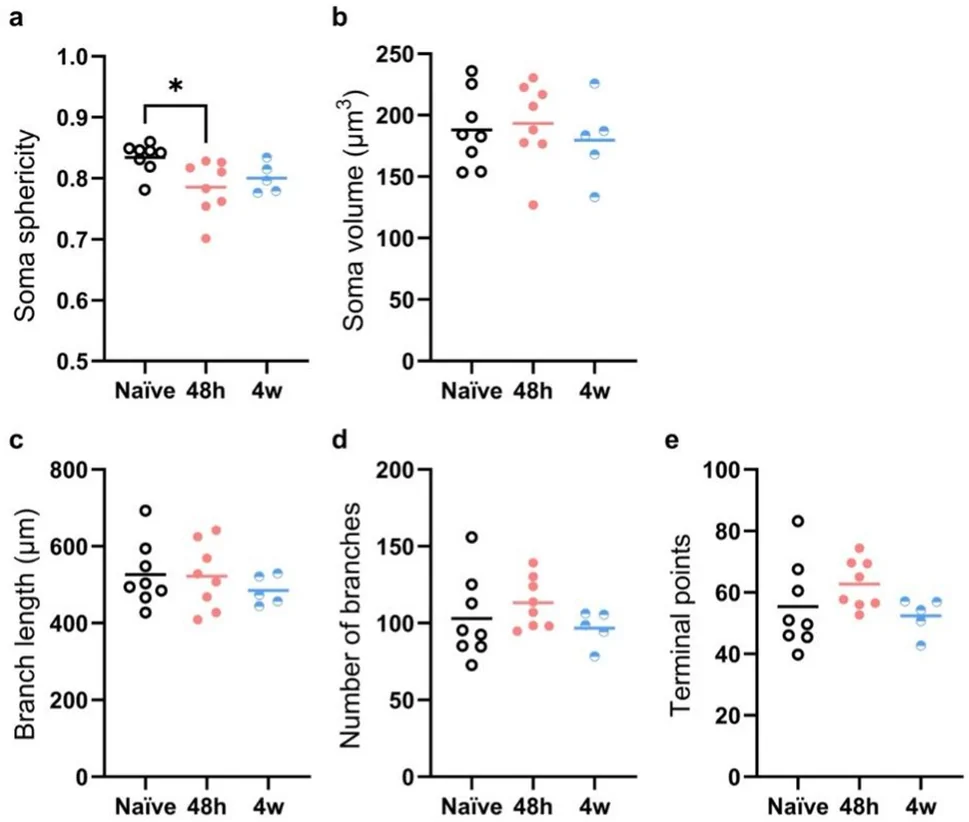
Changes in microglial morphology in the frontal lobe after cardiopulmonary bypass (CPB). Microglial soma sphericity (**a**), soma volume (**b**), branch length (**c**), number of branches (**d**) and number of terminal points (**e**) are shown in naïve sheep (empty black circles; *n* = 8), sheep 48 h after CPB (48 h, red circles; *n* = 8) and 4 weeks after CPB (4w, blue half-filled circles; *n* = 5). Data points are averaged microglia from individual animals; bars represent the mean. Normality was tested using the Shapiro-Wilk test and differences between groups were tested using a one-way ANOVA and between group differences tested using a Dunnett’s multiple comparisons test. *P < 0.05, ***P < 0.001

Representative images for both microglia and astrocytes are provided in Fig. 2. Morphometric analyses of microglia are shown in Figs. 3 and supplementary Figure S1. Compared with naïve controls, the CPB-48 h group exhibited reduced soma sphericity (0.83 ± 0.02 vs. 0.79 ± 0.04, *P* = 0.018; Fig. 3a). There were no differences between the naïve and CPB-4w groups (0.80 ± 0.02, *P* = 0.16) in soma sphericity. Other morphological measures, including branch parameters and Sholl analysis, showed no differences between groups.

**Fig. 4:**
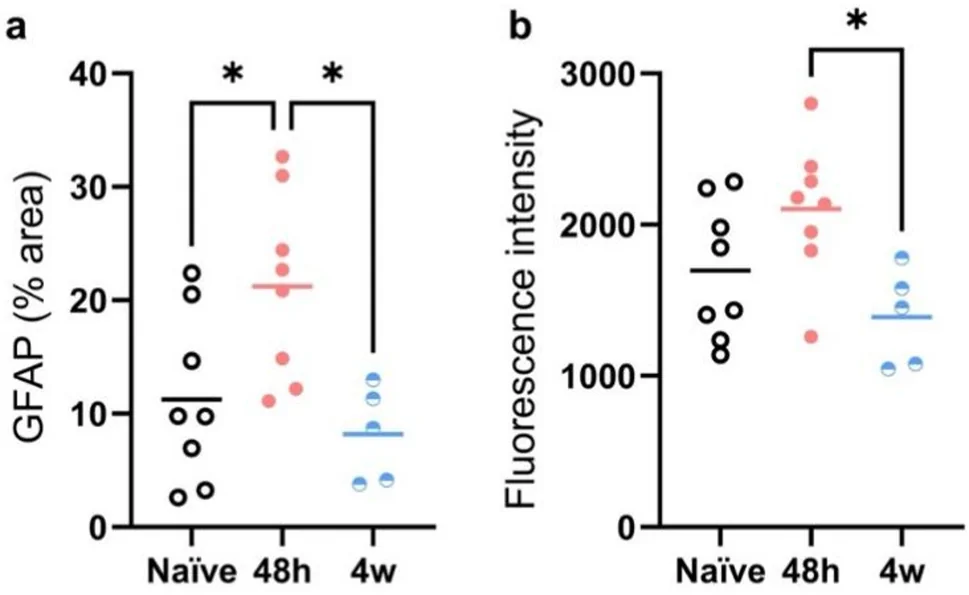
Astrogliosis in the frontal lobe after cardiopulmonary bypass (CPB). Astrocyte reactivity was determined by assessing (**a**) glial fibrillary associated protein (GFAP)-stained area, expressed as percentage area (% area) and (**b**) fluorescence intensity in naïve sheep (empty black circles; *n* = 8), sheep 48 h after CPB (48 h, red circles; *n* = 8) and 4 weeks after CPB (4w, blue half-filled circles; *n* = 5). Data points are individual animals; bars represent the mean. Normality was tested using the Shapiro-Wilk test and differences between groups were tested using a one-way ANOVA and between group differences tested using a Dunnett’s multiple comparisons test. *P ≤ 0.05

Quantitative analyses of astrocytes are presented in Fig. 4. Compared with the naïve controls, the CPB-48 h group exhibited increased GFAP-stained area (11 ± 7% vs. 21 ± 8%, *P* = 0.030; Fig. 4a). Fluorescence intensity was not significantly different between the two groups (*P* = 0.16). There were also significant differences in GFAP-stained area (21 ± 8% vs. 8 ± 4%, *P* = 0.013) and fluorescence intensity (2103 ± 450 vs. 1388 ± 319, *p* = 0.022) between the CPB-48 h and CPB-4w groups. No significant difference was observed between the naïve and CPB-4w groups.

### Systemic haemodynamics

Compared with pre-CPB baseline, during CPB MAP and arterial oxygen content were significantly reduced (Supplementary Table S1). In the first 48 h (Table 1, Supplementary Table S2), MAP was significantly reduced at 12 h (88.9 ± 10.6 vs. 69.7 ± 8.7, *P* = 0.001) and 24 h (79.9 ± 7.5 mmHg, *P* = 0.021) but returned to baseline by 48 h (82.8 ± 7.4 mmHg, *P* = 0.074). Arterial oxygen content remained lower at all timepoints relative to baseline. Systemic oxygen delivery, consumption and oxygen extraction ratio did not change significantly. From 1 to 4 weeks (Table 2 and Supplementary Table S3), MAP remained stable without significant temporal changes over time.

**Table 1:** Cerebral and systemic variables in the immediate post-CPB period (48 h)

Variable	*N*	Pre-CPB Baseline	12 h	24 h	48 h	*P*_time_
Cerebral tPO_2_ (mmHg)	13	24.1 [20.9, 26.7]	13.3 [10.8, 25.2]	16.9 [11.2, 27.6]	18.6 [15.2, 30.6]	0.12
Cerebral tissue perfusion (BPU)	10	533 [463, 699]	531 [439, 713]	561 [450, 863]	597 [473, 862]	0.67
Mean arterial pressure (mmHg)	16	88.9 ± 10.6	69.7 ± 8.7***	79.9 ± 7.5*	82.8 ± 7.4	< 0.0001
Cardiac output (mL/kg/min)	11	NA	123 [116, 137]	131 [110, 136]	108 [100, 139]	0.16

Data are expressed as mean ± SD or median [Q1, Q3], depending on the distribution (assessed by the Shapiro-Wilk test). Sample size is indicated on each variable, as some of the data are missing due to equipment failure. P values (P_time_) were derived from mixed-effected modelling with a Greenhouse–Geisser correction or Friedman test for the main effect of time, as appropriate. When statistical significance was reached (P_time_ < 0.05), within-animal comparisons with pre-CPB baseline were conducted using Dunnett’s test or Dunn’s test (*P ≤ 0.05, ***P ≤ 0.001). CPB; cardiopulmonary bypass, NA; not available

**Table 2:** Systemic and cerebral variables from one to four weeks post-CPB

Variable	*N*	Pre-CPB Baseline	1 week	2 weeks	3 weeks	4 weeks	*P*_time_
Cerebral tPO_2_ (mmHg)	6	21.6 ± 8.2	20.9 ± 7.7	21.8 ± 7.3	25.3 ± 11.1	25.1 ± 2.7	0.77
Cerebral tissue perfusion (BPU)	5	681 ± 416	454 ± 108	438 ± 174	312 ± 119	522 ± 261	0.32
Mean arterial pressure (mmHg)	8	90.4 ± 13.0	84.5 ± 7.1	87.3 ± 10.3	84.5 ± 5.1	85.3 ± 8.6	0.38
Cardiac output (mL/kg/min)	5	NA	132 ± 16.8	120 ± 9.86	125 ± 11.5	110 ± 15.1	0.036

Data are presented as mean ± SD. (Distribution was assessed using the Shapiro–Wilk test.) Sample size is indicated on each variable, as some of the data are missing due to equipment failure. P values (P_time_) were derived from mixed-effects modelling with a Greenhouse–Geisser correction assessing the main effect of time. CPB; cardiopulmonary bypass, NA; not available

### Cerebral oxygenation and perfusion

During CPB, cerebral tPO_2_ and perfusion did not differ significantly from baseline (Table S1). Similarly, cerebral tPO_2_ and perfusion did not change significantly during the first 48 h post-CPB (Table 1). From 1 to 4 weeks post-CPB, cerebral tPO_2_ and perfusion also remained stable, with no significant changes (Table 2).

### Plasma biomarkers of brain injury and systemic inflammation

**Fig. 5:**
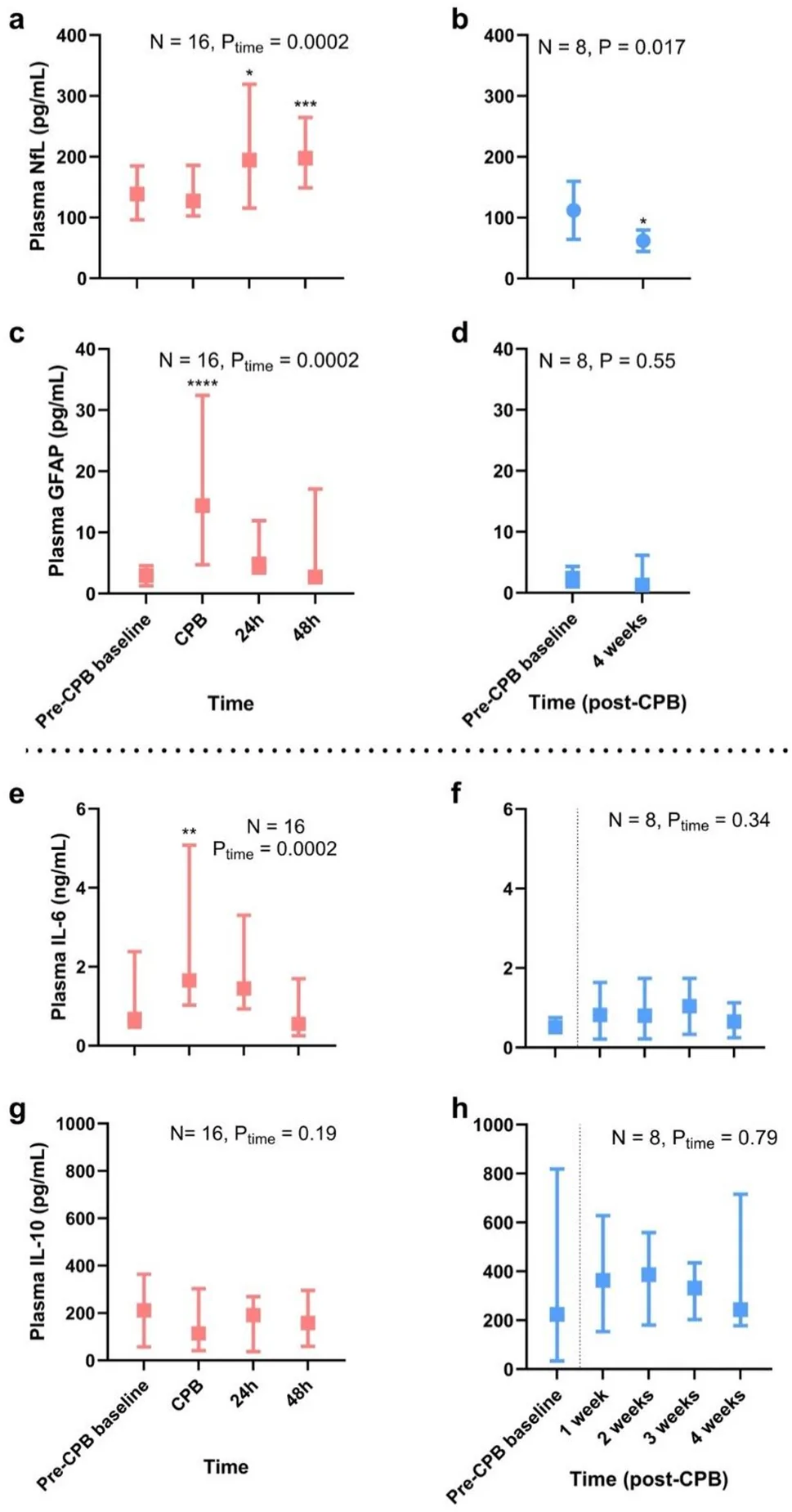
Plasma biomarkers. (**a**, **b**) Neurofilament light chain (NfL), (**c**, **d**) glial fibrillary acidic protein (GFAP), (**e**, **f**) interleukin (IL)-6, and (g, h) IL-10. Data are presented as median (square) with interquartile range [Q1, Q3] or as mean (circle) ± SD, as appropriate (distribution assessed by the Shapiro–Wilk test). P values were derived from the Friedman test, paired t-test, or Wilcoxon matched-pairs test. For the immediate postoperative period (within 48 h), when statistical significance was reached (*P* ≤ 0.05), within-animal comparisons across time points were performed using Dunn’s post hoc test. *P ≤ 0.05, **P ≤ 0.01, ****P ≤ 0.0001, compared with pre-CPB baseline. CPB: cardiopulmonary bypass

In the first 48 h post-CPB, plasma levels of NfL increased over time (P_time_ = 0.0002; Fig. 5a). NfL levels were 138 [96, 185] pg/mL at baseline, and were significantly higher at 24-h (195 [115, 319] pg/mL, *P* = 0.023) and at 48 h (198 [149, 265] pg/mL, *P* = 0.0007). GFAP levels also increased significantly over time (P_time_ = 0.0002; Fig. 5c). GFAP levels increased from 3.0 [1.3, 4.5] at baseline to a peak at the end of CPB (14.4 [4.7, 32.4] pg/mL, *P* < 0.0001), followed by a gradual decline. By 48-h, GFAP levels (2.7 [1.8, 17.1] pg/mL) were not significantly different from baseline (*P* = 0.82). At 4 weeks, NfL levels (62 ± 18 pg/mL, *P* = 0.017) were less than pre-CPB baseline (112 ± 48 pg/mL; Fig. 5b), whereas GFAP levels (1.3 [0.8, 6.2] pg/mL, *P* = 0.55) did not differ significantly from baseline (2.4 [1.0, 4.3] pg/mL; Fig. 5d).

Plasma levels of IL-6 increased in the first 48-h post-CPB (P_time_ = 0.0002). IL-6 increased from 0.68 [0.47, 2.38] ng/mL at pre-CPB baseline to a peak of 1.65 [1.03, 5.08] ng/mL (*P* = 0.0062) at CPB, followed by a decline to 0.55 [0.25, 1.70] ng/mL at 48-h post-CPB. IL-10 did not alter significantly during this period (P_time_ = 0.19). From one to four weeks post-CPB, neither IL-6 nor IL-10 levels differed significantly from pre-CPB baseline values.

## Discussion

### Key findings

In this large mammalian model of CPB, we identified evidence of neuroinflammation in the frontal cortex at 48 h, characterised by astrogliosis and partial microglial activation. These changes occurred despite preserved cerebral tissue oxygenation during and after CPB. This CPB-induced neuroinflammatory response appeared transient and was not seen in animals euthanised 4 weeks postoperatively. Importantly, histological evidence of astrogliosis and partial microglial activation was observed alongside temporal changes in circulating GFAP during CPB and NfL at 48 h. While these changes are temporally associated, the study was not designed or powered to determine diagnostic accuracy or clinical utility of these biomarkers.

### Relationships with previous studies

We did not observe frontal cortical tissue hypoxia or hypoperfusion during CPB, consistent with our previous findings [10, 11]. In clinical studies, cerebral desaturation, measured by NIRS, has been reported in 50–70% of patients [34–36]. However, NIRS only measures cerebral oxygenation indirectly, and can be affected by haemodilution and extracerebral tissue signals [12]. By extending our observations into the postoperative period, we demonstrated that frontal cortical tissue oxygenation and perfusion were maintained for up to 4 weeks after CPB. Our findings suggest that, at least in healthy sheep, cerebral tissue oxygenation is well maintained during CPB under currently recommended clinical perfusion conditions [28]. 

Our findings support the notion that CPB-associated neurological complications can arise independently from sterile inflammatory responses within the brain. We previously reported an exaggerated release of pro-inflammatory cytokines in the blood which was associated with microglial activation across several cortical regions of the brain, with the frontal cortex being the most susceptible to neuroinflammation by the end of CPB in sheep [13]. Similar findings have been reported in other preclinical CPB models [37, 38]. In this study, we observed morphometrical changes in microglia in the frontal cortex at 48 h after CPB, characterised by significantly decreased soma sphericity. This feature is consistent with an activated state of microglia, although the absence of changes in branch metrics and other parameters suggests that they may not be fully activated or, alternatively, may be representative of a reparative process in the aftermath of CPB-related injury. Neuroinflammation after CPB are thought to be initiated by the exposure of blood to foreign surfaces within the CPB circuit, leading to increases in circulating pro-inflammatory cytokines and a disruption of the BBB that causes these mediators to enter the central nervous system [39]. Supporting this, the BBB disruption after CPB has been demonstrated in MRI studies [40, 41] and by the presence of albumin in the cerebrospinal fluid [42] in patients. Notably, the attenuated neuroinflammatory phenotype observed at 48 h, compared with the pronounced responses reported immediately after CPB [13], mirrors the systemic inflammatory trajectory observed in this study, in which plasma IL-6 peaked at the end of CPB and subsequently declined in the postoperative period. This temporal alignment supports a potential link between systemic inflammation and central neuroinflammatory processes.

We also observed astrogliosis at 48 h after CPB, characterised by increased GFAP-stained area. Astrocytes are key modulators of the response to inflammatory triggers and astrogliosis is a hallmark of brain injury, as shown in neurodegenerative diseases and traumatic brain injury [16, 43]. No previous studies have directly assessed the effects of CPB on astrocytes. By 4 weeks post-CPB, the GFAP-stained area had normalised to levels observed in naïve sheep, indicating the CPB-associated neuroinflammatory response is transient in young, healthy animals, and does not produce lasting alterations in the gross morphology of microglia or astrocytes. However, further investigation is needed to determine whether more subtle or persistent molecular changes occur within these glial populations and whether deeper regions of the brain are more vulnerable to persistent changes. Moreover, it remains an important question whether this apparent resolution is maintained in the context of aging or pre-existing comorbidities.

In cardiac surgery, postoperative increases in blood GFAP have been reported in a few clinical studies [20, 21], but these studies measured GFAP either intraoperatively or only immediately after surgery, and their clinical significance remains unclear. The mechanisms underlying the discrepancy between sustained astrogliosis at 48 h and the normalisation of plasma GFAP requires further investigation. This may reflect differing dynamics between astrogliosis and the release of GFAP into circulation.

In contrast to GFAP, plasma NfL continued to rise over the 48 h post-CPB. Postoperative increases in NfL levels are ubiquitous after cardiac surgery [17–19], with greater increases in on-pump than off-pump procedures [17]. However, its clinical utility is uncertain. In a nested study within an randomised clinical trial of patients undergoing on-pump surgery, preoperative NfL levels but not postoperative increases at day 1 were associated with delirium [18]. Subsequent analysis of the same trial reported the link between postoperative increases in NfL and greater cognitive decline at 1 year after surgery [19]. Importantly, GFAP provides a direct measure of astrogliosis, whereas NfL reflects axonal injury and does not serve as a glial injury marker. Further research is needed to determine whether the magnitude of postoperative NfL elevation correlates with histopathological evidence of neuronal injury after CPB, and whether these biomarkers may help evaluate targeted interventions to reduce CPB-associated brain injury.

### Study implications

Our findings indicate that, under currently recommended perfusion strategies, preserving cerebral tissue oxygenation and perfusion does not prevent neuroinflammation and therefore brain injury. CPB-related astrogliosis persists for at least 48 h postoperatively, suggesting that sustained neuroinflammation could contribute to postoperative neurological complications. These observations support targeting neuroinflammatory pathways as a strategy to mitigate CPB-associated neurological disorders. Of particular interest is the relationship between neuroinflammation and circulating biomarkers. GFAP peaked at the end of CPB, consistent with its role as an early indicator of astrogliosis, whereas NfL showed a more prolonged elevation, potentially reflecting ongoing or delayed axonal injury. These findings support the presence of neuroinflammatory responses following CPB despite preserved cerebral oxygenation and generate hypotheses regarding the relationship between tissue-level neuroinflammation and circulating biomarkers. Further adequately powered studies are required to determine whether NfL and GFAP reliably reflect perioperative brain injury in this setting.

### Strengths and limitations

Our study has several strengths. Firstly, we conducted a comprehensive assessment of changes in systemic haemodynamics, cerebral microcirculation, neuroinflammation and blood biomarkers of brain injury. To our knowledge, this is the first study to characterise post-CPB changes in microglia and astrocytes along with relevant blood biomarkers over an extended period. Our model replicates key aspects of human CPB, including pump flow, perfusion pressure, temperature and anticoagulation managements, the choice of anaesthetic drugs, and a 2-h period of aortic cross-clamping with cardioplegic arrest [28, 44], ensuring its strong clinical relevance. The experimental protocols were co-designed and implemented with practicing clinicians (cardiac anaesthesiologists, intensivists, cardiac surgeons and clinical perfusionists) to ensure experimental protocols were as consistent as possible with current practice. Additionally, we assessed cerebral tissue oxygenation using direct measurements, thus avoiding limitations of NIRS measurements, including the effects of haemodilution and contamination from the extracerebral tissues [12]. 

We acknowledge some limitations. First, we used young, healthy sheep, which do not fully represent the heterogeneous clinical population undergoing CPB. This likely attenuated observed abnormality or injury, and the clinical impact of CPB is probably more pronounced. Second, we did not perform cognitive assessments in our animals, as they would be challenging in the highly instrumented sheep. Future longitudinal studies with more frequent sampling are required to better define the kinetics of NfL and GFAP, and their relationship to neuroinflammation and neurological functional outcomes [45]. Third, postoperative analgesia included administration of flunixin meglumine, a non-steroidal anti-inflammatory drug, which may have attenuated components of the peripheral and brain tissue inflammatory responses. This was implemented as part of routine animal welfare requirements. While this approach aligns with ethical standards for large animal experimentation, it may have modulated the magnitude of CPB-associated inflammatory changes. Future studies designed to specifically interrogate neuroinflammation following CPB should consider analgesic strategies that minimise anti-inflammatory confounding, for example utilising opioid-based analgesia alone where ethically and scientifically appropriate. Fourth, our ovine model was designed to investigate the effects of CPB and aortic cross-clamping and did not include a cardiac surgical procedure. Consequently, the inflammatory responses observed in this study may not fully reflect the magnitude or complexity of neuroinflammation encountered following clinical cardiac surgery, where additional inflammatory stimuli arise from surgical tissue injury and perioperative factors. However, this preclinical model was intentionally employed to dissociate the contribution of CPB itself to postoperative neuroinflammatory responses, demonstrating that CPB alone is sufficient to induce sustained neuroinflammation in the frontal cortex. Fifth, we used only female sheep, because handling more aggressive rams poses occupational risks, and chronic bladder catheterisation for the perioperative management is not feasible in male sheep [22]. Sixth, due to technical issues in tissue preservation, only five brains from the CPB-4w group were included for immunohistochemical analyses, which reduced the statistical power to detect more subtle long-term histological changes. Finally, we accessed the heart via a right lateral thoracotomy, as systemic haemodynamics are compromised in the dorsal recumbency required for median sternotomy [40]. 

## Conclusions

In a clinically relevant, large mammalian model of CPB, we demonstrated astrogliosis and partial microglial activation in the frontal cortex at 48 h, which resolved by 4 weeks post-CPB, occurring despite preserved tissue oxygenation during and after CPB. These findings confirm the presence of a neuroinflammatory response to CPB independent of overt hypoxic injury. The association of frontal cortical tissue neuroinflammatory changes with elevations in plasma NfL and GFAP suggests their potential utility as biomarkers of postoperative brain injury and therefore warrants further investigation.

## Supplementary Information

Supplementary Material 1.

## Data Availability

The datasets used and/or analysed during the current study are available from the corresponding author on reasonable request.
