# Supplementary material for: Frontal cortical neuroinflammation and circulating biomarkers following cardiopulmonary bypass in sheep
Source: Intensive Care Med Exp. 2026 Oct 10;14:134. doi: 10.1186/s40635-026-00986-2 (PMC13653855; doi:10.1186/s40635-026-00986-2)
Supplement: Supplementary file 1 [file 40635_2026_986_MOESM1_ESM.pdf]

Original Investigation

Frontal Cortical Neuroinflammation and Circulating Biomarkers Following Cardiopulmonary Bypass in Sheep

Additional File 1

Supplementary Material

Supplementary Methods

Animal care and monitoring

All animals were monitored by researchers and animal technicians daily after the preparatory surgeries through to the completion of the protocol. Predefined ethical criteria for euthanasia included the cessation to eat for four days or drink for two days; malaise or muscle weakness unresponsive to supportive treatment and injury or dysfunction of the carotid artery loop. For surgical prophylaxis, sheep received intramuscular injections of procaine penicillin (900 mg; Ilium®, Troy Laboratories, Australia) for two days postoperatively and intravenous cefazolin (1 g) at 4 and 16 h post-CPB.

Surgical preparation

All animals were acclimatised to the laboratory environment for one week prior to experimentation. They were housed in individual metabolic cages with unrestricted access to water and 800 g of oaten chaff per day. Sheep allocated to the CPB-48h and CPB-4w groups underwent two aseptic surgical procedures under general anaesthesia with isoflurane. In the first procedure, bilateral carotid arterial loops were constructed to facilitate arterial cannulation[1], as peripheral arterial access (e.g., radial or femoral arteries) is not readily feasible in sheep. Three to four weeks later, a carotid artery and jugular vein were cannulated, and a second surgical procedure was performed to implant custom-made combination fibre-optic probes (Oxford Optronix, Abingdon, United Kingdom) into the frontal cortex.[2] Animals were allowed at least 5 days recovery following the second procedure prior to the CPB experiment.

Cardiopulmonary bypass

After a right thoracotomy was performed to access the heart, a transit-time flow probe (20 mm, Transonic Systems, New York, United States) was placed around the pulmonary artery to measure cardiac output. Unfractionated heparin was administered intravenously (300 IU kg^{-1}, Pfizer, NSW, Australia), and an arterial cannula (22 or 24 Fr, EOPA77424, Medtronic, MN,

United States) and a single stage venous cannula (34 Fr, DPL66132, Medtronic, MN, United States) were inserted into the ascending aorta and right atrium, respectively. A combination aortic root cannula was inserted to deliver antegrade cardioplegia and was used subsequently as a left ventricular vent.

A Stockert S3 heart-lung machine with a hollow fibre membrane oxygenator and integrated arterial filter (Sorin 6F, LivaNova, Vic, Australia) was used. The tubing pack was designed for sheep (Softcell, LivaNova, Vic, Australia). The perfusion circuit was primed with 1 g of cefazolin (AFT Pharmaceuticals, NSW, Australia), 50 mL of mannitol (20% w/v Osmitrol, Baxter, NSW, Australia) and 10,000 IU of heparin, with the volume adjusted to 1.3 L using balanced crystalloid (PlasmaLyte®, Baxter, NSW, Australia).

CPB was commenced with a target non-pulsatile flow of 2.4 L min^{-1} m^{-2}, with body surface area calculated according to Bennett.[3] The oxygenator gas flow was set at 1,200 mL min^{-1} with 60% oxygen and adjusted to target a PCO_2 of 35–45 mmHg. The target body temperature (measured in the kidney) was 36 °C (the normal ovine body temperature being 39 °C). The target mean arterial pressure (MAP) was 65–75 mmHg. Intravenous boluses or a continuous infusion of metaraminol (Montrose Life Sciences, NSW, Australia) were used as required to maintain target MAP. If MAP could not be maintained ≥ 70 mmHg with vasopressors, or if mixed venous SO_2 was < 70%, the pump flow was increased by 10%.

The aorta was cross-clamped and anterograde blood cardioplegia (4:1) was administered for myocardial protection; an initial dose of 1000 mL (AHK5534®, Baxter, NSW, Australia) was followed by 300 mL cardioplegia (AHK5535®, Baxter, NSW, Australia) at 15-min intervals until the aortic cross-clamp was removed.

During the final 15 min of the 2-hour period of aortic cross-cramp, animals were rewarmed to 39 °C. The aortic cross-clamp was then removed. The animal was weaned off CPB, protamine

was administered (3 mg kg^{-1}, Fisons Protamine Sulfate, Sanofi, NSW, Australia) and the aortic, right atrial and aortic root cannulas were removed. Residual blood from the CPB circuit was infused to optimise volume status. After decannulation and chest closure, anaesthesia was discontinued, animals were extubated when clinically appropriate and then returned to individual metabolic cages for postoperative observation.

Experimental Measurements

Total peripheral conductance was determined by dividing cardiac output (CO) (or pump flow during CPB) by the product of mean arterial pressure (MAP) and body weight. Blood samples were collected via arterial and jugular catheters for blood oximetry and biochemistry (ABL system 625, Radiometer Medical, Copenhagen, Denmark). Systemic oxygen delivery (DO_2), oxygen consumption (VO_2) and oxygen extraction ratio were calculated according to the following formulae:

$$DO_2 = CO\ (or\ pump\ flow\ during\ CPB) \times Arterial\ O_2\ Content$$

$$VO_2 = CO\ (or\ pump\ flow\ during\ CPB)\ \times (Arterial\ O_2\ Content - Venous\ O_2\ Content)$$

$$O_2\ Extraction\ Ratio = 100 \times (\frac{Arterial\ O_2\ Content - Venous\ O_2\ Content}{Arterial\ O_2 Content})$$

In some animals, physiological recordings were not available because of equipment malfunction (e.g., tissue oxygenation probe failure). These technical issues did not affect animal recovery or tissue collection. Separately, immunohistochemical analyses could not be performed in three brains from the CPB-4w group because tissue preservation was incomplete during post-mortem processing.

Supplementary Figures

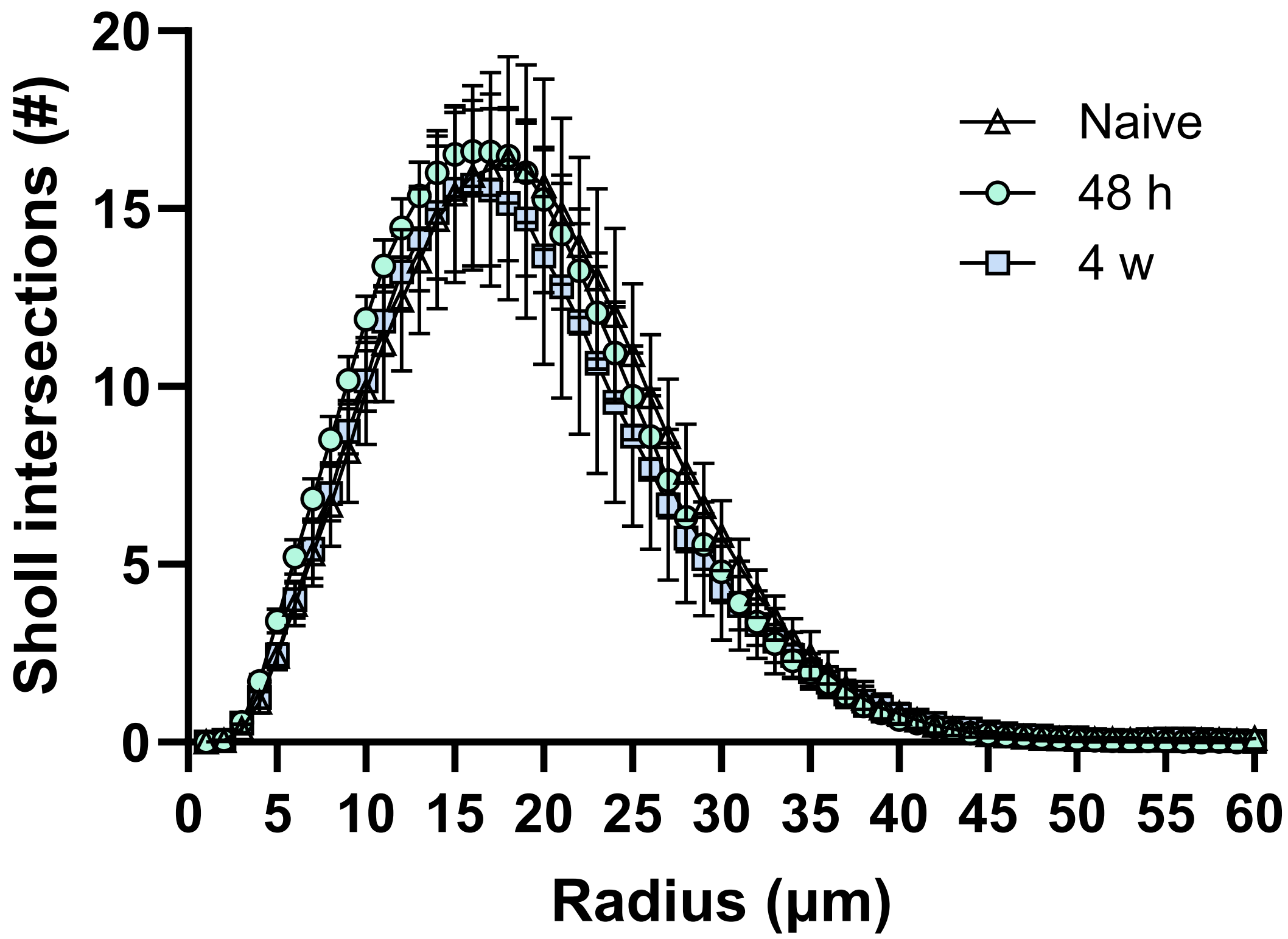

Figure S1. Sholl analysis in microglial morphology in the frontal lobe after cardiopulmonary bypass (CPB).

Supplementary Tables

Table S1. Systemic and cerebral variables during CPB

Variable	N	Pre-CPB Baseline	CPB	P value
Mean arterial pressure (mmHg)	16	88.9 ± 10.6	66.4 ± 4.8	< 0.0001
Total peripheral conductance (mL/kg/min/mmHg)	16	NA	0.99 [0.96, 1.09]	NA
Systemic oxygen delivery (mL O_2/kg/min)	16	NA	6.94 [6.15, 7.40]	NA
Systemic oxygen consumption (mL O_2/kg/min)	16	NA	2.25 ± 0.49	NA
Systemic oxygen extraction (%)	16	32.7 ± 5.0	33.3 ± 6.9	0.77
Body temperature (°C) †	16	39.9 [39.4, 40.4]	36.1 [35.7, 36.5]	< 0.0001
Haemoglobin (g/dL) ††	16	9.33 ± 1.20	6.61 ± 0.65	< 0.0001
Arterial pH	16	7.52 ± 0.03	7.43 ± 0.03	< 0.0001
Arterial SO_2 (%)	16	95.9 ± 1.4	99.0 ± 0.5	< 0.0001
Arterial Oxygen Content (mL O_2/dL)	16	12.7 ± 1.6	10.0 ± 0.9	< 0.0001
Arterial PCO_2 (mmHg)	16	35.3 ± 3.4	40.1 ± 3.5	0.0016
Mixed venous SO_2 (%)	16	66.0 ± 6.1	72.0 ± 7.5	0.0056
Lactate (mmol/L)	16	0.50 [0.50, 0.85]	2.96 [2.36, 3.45]	< 0.0001
Cerebral tPO_2 (mmHg)	13	24.1 [20.9, 26.7]	27.3 [15.9, 41.7]	0.094
Cerebral tissue perfusion (BPU)	12	526 [376, 617]	391 [337, 525]	0.092

Data are presented as mean ± standard deviation (SD) or median [Q1, Q3], depending on distribution, assessed using the Shapiro–Wilk test. Formulas used for the calculation of variables are provided in Supplemental Methods. Sample size is indicated for each variable, as some of the data are missing due to equipment failure. Pre-CPB baseline was recorded in the non-anaesthetised state. For the CPB period, mean arterial pressure, body temperature, cerebral tissue oxygenation and perfusion were averaged over the 2-h CPB period. Other variables were calculated as averages of four 30-minute intervals during CPB. P-values were derived from paired t-tests or Wilcoxon matched-pairs signed-rank tests, as appropriate. †The normal body temperature in sheep is 39–40°C. ††Baseline haemoglobin is lower in sheep (8.5–11 g/dL)[4,5] than humans, and the observed decrease in haemoglobin levels in the experimental animals (approximately 30%) was similar to that seen in humans receiving CPB.[6] CPB; cardiopulmonary bypass, NA; not available.

Table S2 Systemic variables during the initial 48 h post-CPB

Variable	N	Pre-CPB Baseline	12 h	24 h	48 h	P_{time}
Total peripheral conductance (mL/kg/min/mmHg)	11	NA	1.82 ± 0.42	1.65 ± 0.26	1.41 ± 0.24	0.0001
Systemic oxygen delivery (mL O_2/kg/min)	11	NA	10.7 [9.5, 13.1]	11.6 [8.1, 13.1]	10.2 [9.3, 11.2]	0.48
Systemic oxygen consumption (mL O_2/kg/min)	11	NA	3.80 ± 1.38	3.83 ± 0.69	4.29 ± 1.87	0.91
Systemic oxygen extraction (%)	16	32.7 ± 5.0	34.9 ± 10.7	35.4 ± 8.3	36.4 ± 10.1	0.52
Body temperature (°C)	16	39.9 ± 0.66	39.0 ± 0.5***	39.2 ± 0.5**	39.5 ± 0.3*	< 0.0001
Haemoglobin (g/dL) †	16	9.33 ± 1.20	6.17 ± 1.00****	6.57 ± 0.96****	7.29 ± 0.94****	< 0.0001
Arterial pH	16	7.52 ± 0.03	7.52 ± 0.03	7.51 ± 0.04	7.49 ± 0.04	0.035
Arterial SO_2 (%)	16	95.9 ± 1.43	96.4 ± 1.84	95.3 ± 2.3	95.1 ± 2.5	0.039
Arterial PCO_2 (mmHg)	16	35.3 ± 3.4	34.5 ± 2.5	35.9 2.4	34.9 3.0	0.52
Arterial Oxygen Content (mL O_2/dL)	16	12.7 ± 1.55	8.55 ± 1.25****	8.84 ± 1.25****	9.90 ± 1.15****	< 0.0001
Mixed venous SO_2 (%)	16	66.8 [62.7, 70.6]	68.3 [54.1, 70.6]	64.6 [56.3, 69.7]	59.7 [53.9, 67.4]	0.25
Lactate (mmol/L)	16	0.50 [0.40, 0.85]	0.70 [0.60, 0.98]	0.70 [0.40, 1.08]	0.45 [0.33, 0.50]	0.0090

Data are presented as mean ± standard deviation (SD) or median [Q1, Q3], depending on distribution, assessed using the Shapiro–Wilk test. Formulas used for the calculation of variables are provided in Supplemental Methods. P values (P_{time}) were derived from mixed-effects modelling with a Greenhouse–Geisser correction or from Friedman's test with Dunn's correction assessing the main effect of 'time'. When statistical significance was reached ($P_{time} < 0.05$), within-animal comparisons with pre-CPB baseline in the non-anaesthetised state were conducted using Dunnett's test or Dunn's test. *P ≤ 0.05, **P ≤ 0.01, ***P ≤ 0.001, ****P ≤ 0.0001. †Baseline haemoglobin is lower in sheep (8.5–11 g/dL)[4,5] than humans, and the observed decrease in Hb levels in the experimental animals (approximately 30%) was similar to that seen in humans receiving CPB.[6] CPB; cardiopulmonary bypass, NA; not available.

Table S3 Systemic variables in the extended post-CPB period (1 to 4 weeks)

Variable	N	Pre-CPB Baseline	1 week	2 weeks	3 weeks	4 weeks	P_{time}
Total peripheral conductance (mL/kg/min/mmHg)	4	NA	1.57 ± 0.27	1.37 ± 0.10	1.50 ± 0.18	1.29 ± 0.15	0.20
Systemic oxygen delivery (mL O_2/kg/min)	5	NA	10.8 ± 1.2	10.6 ± 1.7	11.4 ± 1.5	9.2 ± 1.8	0.032
Systemic oxygen consumption (mL O_2/kg/min)	5	NA	4.67 ± 1.61	4.15 ± 0.72	5.16 ± 0.72	4.54 ± 0.51	0.39
Systemic oxygen extraction (%)	8	NA	33.8 ± 4.6	42.4 ± 10.1	42.3 ± 10.1	46.6 ± 12.6	0.25
Body temperature (°C)	8	40.1 ± 0.7	40.0 ± 0.3	39.7 ± 0.5	39.6 ± 0.3	39.8 ± 0.4	0.12
Haemoglobin (g/dL)	8	8.90 ± 1.31	6.18 ± 0.39**	6.33 ± 0.64**	6.59 ± 0.57**	6.34 ± 0.69**	0.0002
Arterial pH	8	7.50 ± 0.02	7.51 ± 0.05	7.49 ± 0.03	7.49 ± 0.05	7.45 ± 0.07	0.17
Arterial SO_2 (%)	8	95.7 ± 1.5	95.7 ± 2.7	95.6 ± 2.1	96.2 ± 1.0	96.1 ± 1.7	0.53
Arterial Oxygen Content (mL O_2/dL)	8	12.1 ± 1.73	8.48 ± 1.38**	8.67 ± 0.80**	9.06 ± 0.81**	8.77 ± 1.02**	0.0002
Arterial PCO_2 (mmHg)	8	35.8 ± 3.1	32.1 ± 3.8	35.1 ± 2.0	35.7 ± 2.8	35.3 ± 1.8	0.070
Mixed venous SO_2 (%)	8	63.9 ± 5.7	56.0 ± 8.9	55.7 ± 9.6	55.5 ± 10.8	50.3 ± 12.1	0.15
Lactate (mmol/L)	7	0.40 [0.40, 0.50]	0.50 [0.40, 0.50]	0.60 [0.40, 0.80]	0.40 [0.40, 0.70]	0.40 [0.30, 0.70]	0.67

Data presentation and statistical analysis are as for Table S2. CPB; cardiopulmonary bypass, NA; not available.

References

1. Lankadeva YR, Kosaka J, Evans RG, May CN: An ovine model for studying the pathophysiology of septic acute kidney injury. *Methods Mol Biol* 2018:1717:207-218. doi: 10.1007/978-1-4939-7526-6_16

2. Jufar AH, May CN, Evans RG, et al.: Influence of moderate hypothermia on renal and cerebral haemodynamics and oxygenation during experimental cardiopulmonary bypass in sheep. *Acta Physiol* 2022; 236:e13860. doi: 10.1111/apha.13860

3. Bennett JW: Regional body surface area of sheep. J Agric Sci 1973; 81:429–32. doi: 10.1017/s0021859600086469

4. Jufar AH, May CN, Booth LC, et al.: Effects of dexmedetomidine on kidney and brain tissue microcirculation and histology in ovine cardiopulmonary bypass: A randomised controlled trial. *Anaesthesia* 2023; 78:1481–92. doi: 10.1111/anae.16152

5. Evans RG, Iguchi N, Cochrane AD, et al.: Renal hemodynamics and oxygenation during experimental cardiopulmonary bypass in sheep under total intravenous anesthesia. *Am J Physiology-regulatory Integr Comp Physiology* 2020; 318:R206–13. doi: 10.1152/ajpregu.00290.2019

6. Mazer CD, Whitlock RP, Fergusson DA, et al.: Restrictive or liberal red-cell transfusion for cardiac surgery. *N Engl J Med* 2017; 377:2133–44. doi: 10.1056/nejmoa1711818
